# Surgeon-General of New York

**Published:** 1863-02

**Authors:** 


*Surgeon-General of New York.*—Gov. Seymour has appointed Dr. John V. P. Quackenbush of Albany, Surgeon-General of New York. Dr. Quackenbush is a professor in the Albany Medical College, and has an established reputation not only for professional ability, but also for courtesy and business capacity.

